# Giant Increase of Hardness in Silicon Carbide by Metastable Single Layer Diamond‐Like Coating

**DOI:** 10.1002/advs.202204562

**Published:** 2023-01-04

**Authors:** Martin Rejhon, Xinliu Zhou, Francesco Lavini, Alessandra Zanut, Filip Popovich, Lorenzo Schellack, Lukasz Witek, Paulo Coelho, Jan Kunc, Elisa Riedo

**Affiliations:** ^1^ Department of Chemical and Biomolecular Engineering Tandon School of Engineering New York University Brooklyn NY 11201 USA; ^2^ Division of Biomaterials Department of Molecular Pathobiology New York University College of Dentistry New York NY USA; ^3^ Charles University Faculty of Mathematics and Physics Institute of Physics Ke Karlovu 5, Prague 2 Prague CZ‐121 16 Czech Republic; ^4^ Department of Physics New York University Brooklyn NY 11201 USA

**Keywords:** diamene, epitaxial graphene, SiC, hardness, Young's modulus

## Abstract

Silicon carbide (SiC) is one of the hardest known materials. Its exceptional mechanical properties combined with its high thermal conductivity make it a very attractive material for a variety of technological applications. Recently, it is discovered that two‐layer epitaxial graphene films on SiC can undergo a pressure activated phase transition into a sp^3^ diamene structure at room temperature. Here, it is shown that epitaxial graphene films grown on SiC can increase the hardness of SiC up to 100% at low loads (up to 900 µN), and up to 30% at high loads (10 mN). By using a Berkovich diamond indenter and nanoindentation experiments, it is demonstrated that the 30% increase in hardness is present even for indentations depths of 175 nm, almost three hundred times larger than the graphene film thickness. The experiments also show that the yield point of SiC increases up to 77% when the SiC surface is coated with epitaxial graphene. These improved mechanical properties are explained with the formation of diamene under the indenter's pressure.

## Graphical abstract

This work shows that the yield point of SiC can be increased by 77% by coating SiC with epitaxial graphene. This enhancement is due to a pressure‐induced graphene (sp^2^) ‐diamene (sp^3^) phase transition. The hardness of graphene coated SiC increases by 30% compared to bare SiC, even for indentation depths 300 times larger than the graphene coating thickness.

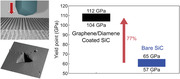

## 1 Introduction

Graphene has shown great potential for mechanical applications due to its exceptional mechanical properties.^[^ ^1^, ^2^, ^3^, ^4^, ^5^ ^]^ Recent investigations of mechanically exfoliated monolayer graphene membranes by atomic force microscopy (AFM) showed extremely high in‐plane stiffness (≈1 TPa) and membrane strength ≈100 GPa.^[^ ^1^ ^]^ However, experiments on graphene grown by chemical vapor deposition (CVD) on a copper substrate showed no increase in hardness of the graphene coated copper substrate.^[^ ^6^ ^]^ Regarding the stiffness of substrates coated with graphene, no increase has been detected for exfoliated graphene on SiO_2_ substrates,^[^ ^7^ ^]^ while a 5% increase has been measured for copper coated with CVD compared to bare copper.^[^ ^6^ ^]^ For a copper substrate coated with graphene, a mild increase in bearing capacity has been shown during the initial elastic regime at low loads.^[^ ^8^ ^]^

Very recently it has been reported that two‐layer epitaxial graphene grown on silicon carbide (SiC) can undergo a room temperature, pressure activated phase transition from sp^2^ to sp^3^ hybridization. This phase transition dramatically changes the elastic properties of the graphene/SiC system under pressure.^[^ ^7^, ^9^, ^10^, ^11^ ^]^ In particular, purely elastic ångström indentation (Å‐indentation) measurements indicated a surface stiffness comparable to diamond, while no investigations on the plastic behavior have been performed.^[^ ^7^, ^9^, ^10^, ^12^ ^]^

**Figure 1:**
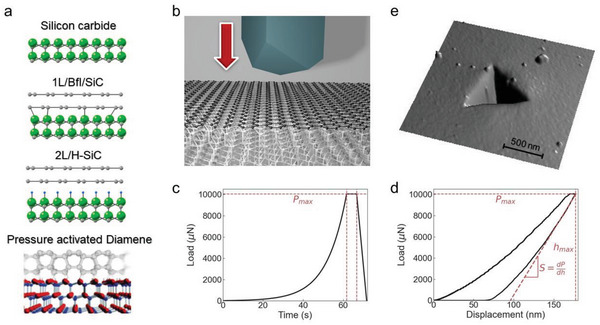
Nanoindentation experiment. a) Schemes of the investigated samples, namely bare silicon carbide, one epitaxial graphene on the carbon buffer layer on silicon face of SiC, and quasi free‐standing bilayer graphene on silicon face of SiC after hydrogen intercalation, and the scheme of pressure activated diamene structure.^[^ ^9^, ^11^ ^]^ b) Scheme of the nanoindentation experiments with a diamond Berkovich tip. c) Nanoindentation experiment consists of three stages, first loading part with the constant strain rate, second stage when the maximal load is held and the third unloading stage while the load is linearly decreased. d) The obtained load‐displacement curve from the nanoindentation experiment. e) Residual imprint on the surface after nanoindentation experiment.

Silicon carbide is considered one of the best materials for protection against high‐speed impacts and for body armor applications^[^ ^13^ ^]^ due to its extreme hardness, strength, and high thermal conductivity. Considering the technological interest in the exceptional mechanical properties of SiC, second only to diamond, here, we investigate how the hardness of a SiC substrate can be further improved when SiC is coated with an atomically thin and thermally conductive epitaxial graphene film. In particular, we conduct Berkovich hardness indentation tests on monolayer (1L) epitaxial graphene films grown on the Si‐face of SiC(0001),^[^ ^14^ ^]^ where a typical buffer carbon layer (BfL) sits in between graphene and SiC^[^ ^14^ ^]^ (see 1L/BfL/SiC in **Figure** **1**a); in addition, we investigate H‐terminated SiC(0001) coated with 2L quasi‐free‐standing epitaxial graphene films^[^ ^15^, ^16^ ^]^ (see 2L/H‐SiC in Figure 1a), and finally we compare the results with a bare SiC(0001) substrate, see the Experimental Section for more details on the samples. The experiments show up to 100% increase in hardness, and 80–50% increase in elastic modulus when SiC is coated with 1 L epitaxial graphene plus buffer layer, or with 2 L quasi‐free‐standing graphene compared to bare SiC at low loads (up to 900 µN). This increase levels off to ≈30% when indentation loads are 10 mN, and indentations depths reach 175 nm. Furthermore, we demonstrate that the minimum pressure leading to residual plastic indents, i.e., the yield point, increases by 77% when SiC is coated with epitaxial graphene films. These results can be explained by the pressure activated sp^2^ to sp^3^ phase transition occurring in two‐layer epitaxial graphene on SiC when a Berkovich tip indents the system and forms a diamond layer, called diamene^[^ ^11^ ^]^ (Figure 1a). Importantly, previous Å‐indentation measurements indicated that diamene was ultra‐stiff, however the indentations used to determine the stiffness of diamene were <1 Å.^[^ ^7^, ^9^, ^10^ ^]^ Here, we show that the atomically thin diamene layer can improve the mechanical properties of SiC even at high loads, corresponding to indentations three hundred times larger than the graphene film thickness. Furthermore, this work suggests that the phase transition can occur also over large areas for large indenters, here at least µm^2^ areas. These findings are promising for future applications in high‐impact protective coatings, body armors production, and for use in aeronautics, aerospace, and automobile industry.

## 2 Results and Discussion

**Figure 2:**
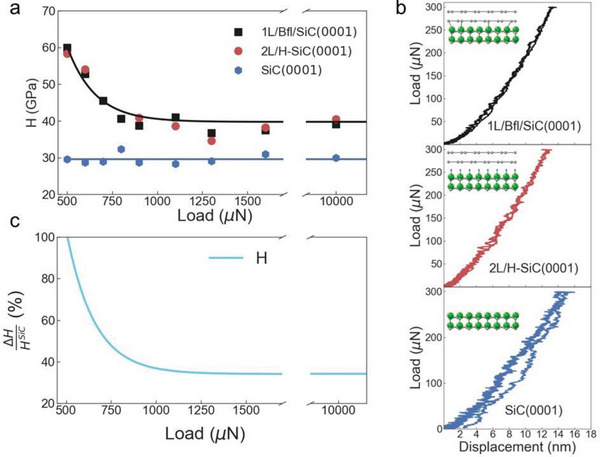
Hardness determined by Oliver‐Pharr method. a) Average hardness dependency on the applied load for different samples. Experimental average data are shown as markers and solid lines represent fit with an exponential decay function as a guide for the eye. b) The load displacement curves at 300 µN showing no plastic deformation in case of SiC samples coated with epitaxial graphene. c) The percentage change in hardness between graphene coated and bare SiC.

The hardness *H* and elastic modulus *E* of bare and graphene coated 6H‐SiC (0001) substrates are investigated by indentation experiments using a Hysitron TI 950 Triboindenter with a diamond Berkovich indenter, applying loads in the range between 300 µN and 10 mN (Figure 1b). During the indentation experiments, the indenter is brought into contact with the surface of the investigated sample while increasing the normal force at a constant rate until a given maximum load is achieved, then the maximum load is held for a certain amount of time, and in the final stage, the load is decreased linearly with time, see Figure 1c. In the experiments, the load and the indentation depth are measured and recorded simultaneously. During the loading part, the Berkovich indenter induces elastic and/or plastic deformations; during the unloading part, the elastic deformation can be fully restored (Figure 1d), therefore a residual imprint of the indenter shape on the sample surface (Figure 1e) remains only if plastic deformations occur during the loading part. The hardness is estimated from the unloading part of the indentation curves using the Oliver–Pharr method^[^ ^17^ ^]^ (see Experimental Section) and plotted as a function of the applied load in **Figure** **2**a. The corresponding load displacement curves are shown in the supplementary information (see Figures S2–S4, Supporting Information). The indenter shape and the indentation projected area needed for the Oliver–Pharr method^[^ ^17^ ^]^ are calibrated based on the known hardness of the bare SiC sample, which is 30 GPa^[^ ^18^, ^19^, ^20^, ^21^ ^]^ (see Experimental Section and Supporting Information). For example, Shaffer et al.^[^ ^21^ ^]^ obtained a hardness of ≈30 GPa along the c‐axis using a Knoop indenter, and Henshall et al.^[^ ^20^ ^]^ obtained hardness values of ≈30 GPa in the (0001) direction using a Berkovich indenter. In Figure 2a, along with the results on bare SiC, we also report the measured hardness of graphene coated SiC samples, namely 1L/BfL/SiC and 2L/H‐SiC (see Figure 1a). The measurements show higher values of hardness in graphene coated SiC compared to bare SiC(0001) in the whole range of investigated loads. Very importantly, at loads lower than 500 µN, the load‐indentation curves do not indicate any plastic deformation in the case of 1L/Bfl/SiC(0001) and 2L/H‐SiC(0001), as can be seen in Figure 2b (more curves can be found in Figures S2–S4, Supporting Information), showing a higher ability of graphene coated SiC to withstand higher pressures before yielding. These results are attributed to the local formation of diamene,^[^ ^9^, ^10^ ^]^ induced by the pressure activated sp^2^ to sp^3^ phase transition in the two graphitic layers under the indenter. This 2D diamond‐like structure on SiC increases the resistance of SiC to yield compared to bare SiC, resulting in higher hardness. On the other hand, previous theoretical work^[^ ^9^ ^]^ predicts no graphene‐diamene phase transition at room temperature for >3 L. Indeed, we find that when SiC is coated with 10 L of epitaxial graphene, the hardness decreases of ≈88% compared to bare SiC. This is also in agreement with previous studies that did not show any improvement in the hardness of copper when coated with only one layer of CVD graphene.^[^ ^6^ ^]^ The increased hardness of the 1L/Bfl/SiC(0001) system is consistent with a coating harder than the SiC substrate.^[^ ^22^ ^]^ The hardness of the graphene/SiC composite structure decreases with increasing loads, reaching a plateau value of ≈40 GPa at 10 mN loads, a value which is 30% larger than the hardness of bare SiC at the same load and closer to the hardness of diamond, i.e., 60–150 GPa.^[^ ^23^ ^]^ Furthermore, compared to bare SiC, the hardness of 1L/Bfl/SiC(0001) and 2L/H‐SiC(0001) at lower loads, e.g. 500 µN, displays an incredible increase of up to 100%, compared to bare SiC, reaching values of 60 GPa (see Figure 2c). Importantly, previous experiments indicated that diamene was ultra‐stiff; however, the indentations used to determine the stiffness of diamene were <1 Å. On the other hand, here, we show that the atomically thin diamene layer can improve the mechanical properties of SiC even at high loads, corresponding to indentations of ≈175 nm, i.e., almost three hundred times larger than the graphene film thickness. Furthermore, the measurements show improved hardness and stiffness even when using a large Berkovich indenter, as opposed to a 10 nm AFM indenter used in previous studies,^[^ ^9^, ^10^ ^]^ suggesting that the phase transition can occur over large areas, in this work areas up to µm^2^ have been probed, as shown in Figure 1e.

**Figure 3:**
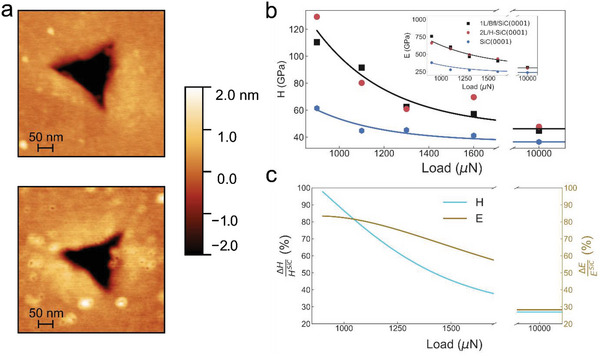
Hardness and Young's modulus determined by AFM imaging of residual indentations. a) Images of residual indentations on the surface of a bare SiC sample and 2L/H‐SiC sample for a load of 1600 µN. b) Hardness evaluated from the AFM topographical images of residual indentations for different samples in the range between 900 and 1600 µN, and 10 mN. The determined Young's modulus is shown in the inset. Experimental data are shown as markers and solid lines represent fit with an exponential decay function as a guide for the eye. c) The percentage change in hardness (blue line) and Young's modulus (gold line) between graphene coated and bare SiC.

The hardness values presented in Figure 2 have been obtained using the Oliver–Pharr method, to confirm these results, we also perform AFM measurements of the projected area of the residual indentations at selected loads of 900, 1100, 1300, 1600, and 10 mN (see **Figure** **3**a). In particular, in the range between 900 and 1600 µN, for which the projected area of the residual indentations is well defined in the AFM maps, we evaluate hardness and elastic modulus for all samples (see Figure 3b,c). By comparing the results in Figures 2, and 3 it is clear that the two methods give consistent hardness results. Regarding the elastic modulus, we find that the Young's modulus of SiC coated with epitaxial graphene is ≈750 GPa when the load is 900 µN, while at the same load the Young's modulus of bare SiC is 400 GPa, in agreement with values reported in literature.^[^ ^19^, ^24^ ^]^ At larger loads, the difference between bare and graphene coated SiC decreases, showing only a 30% increase at 10 mN. The large increase in indentation elastic modulus up to ≈0.7 TPa for graphene coated SiC, at loads as large as 1 mN, is quite surprising considering that at these loads the indenter penetrates 30–40 nm, a depth ≈60 times larger than the thickness of the graphene film. To investigate the stress distribution in the samples, we conducted finite element simulations of the plastic deformation of bare silicon carbide and silicon carbide covered with a five angstroms thick diamond film; the results are reported in Figure S12 (Supporting Information).

**Figure 4:**
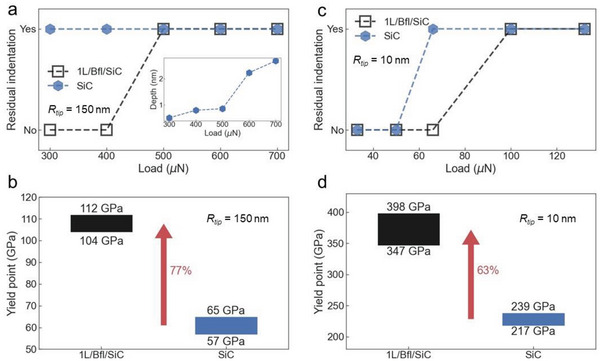
Onset of plastic deformation and yield point. a) Presence of residual indentations after triboindentation tests with Berkovich tip (*R* = 150 nm) on bare SiC(0001) and 1L/Bfl/SiC(0001) samples in the applied load range between 300 and 700 µN. b) Calculated yield point from the Figure 4a showing an increase of ≈77% for 1L/Bfl/SiC sample compared to bare SiC. c) Presence of residual indentations after nanoindentation tests with a diamond AFM tip with radius of 10 nm in the range of applied load between 35 and 140 µN. d) Corresponding yield point for AFM nanoindentation tests showing an increase of ≈63% for 1L/Bfl/SiC sample compared to bare SiC.

Furthermore, we investigate the presence of residual indentations at low loads (300–700 µN) on the surface of bare SiC(0001) and graphene coated SiC to determine the onset of plastic deformation. Interestingly, we observe residual indentations on the surface of bare SiC for all loads, while no residual indentations are present on the surface of 1L/Bfl/SiC at loads 300 and 400 µN (see **Figure** **4**a), in perfect agreement with the behavior of the load displacement curves (see Figure 2b). The first residual indentation is visible on the surface of 1L/Bfl/SiC only after indentation experiments at 500 µN. Figure 4b shows the corresponding calculated yield points *Y*, defined as the pressure at which the sample starts to undergo a plastic deformation, and obtained from the values of the maximum load without a residual indentation and the minimum load with a residual indentation on the sample's surface using the following equation:

(1) $$Y=\frac{1}{\pi }\;{\left(\frac{6F_{Y}E_{r}^{2}}{R^{2}}\right)}^{\frac{1}{3}}$$

where *R* is the radius of the indenter and *E*_r_ is the relative elastic modulus. The yield point varies between the 57 GPa and 65 GPa for bare SiC, while for graphene coated SiC, the yield point is between 104 and 112 GPa, corresponding to an astonishing increase of ≈77% compared to bare SiC. To study the role of the indent size, we perform nanoindentation experiments when the sample surface is indented with a diamond AFM tip with a radius of 10 nm. Figure 4c shows that for this indenter size, residual indentations appear at 58 ± 8 µN for bare SiC, while the 1L/Bfl/SiC sample begins to yield at 83 ± 17 µN, resulting into a yield point between 217 and 239 GPa for bare SiC, and between 347 and 398 GPa for 1L/Bfl/SiC. The values of yield point obtained with a smaller indenter (10 nm) are significantly higher than those obtained with a larger indenter (150 nm), in agreement with previous studies,^[^ ^25^ ^]^ this effect may be driven by a difference in the gradient of deformation outside the indenters, and the need for more geometrically necessary dislocations in the case of sharper indenters.^[^ ^26^ ^]^ However, for both indenter sizes the percentage increase in yield point for 1L/Bfl/SiC compared to bare SiC exhibits similar values, specifically 77% for the triboindenter experiment (150 nm radius), and 63% for the AFM nanoindentation experiment (10 nm).

**Figure 5:**
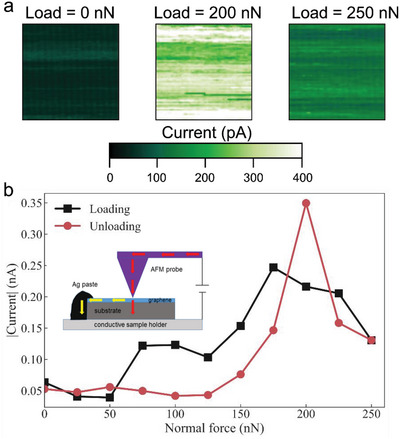
C‐AFM measurements. a) c‐AFM maps taken at loads of 0, 200, and 250 nN on a 1L/Bfl/SiC(0001) sample with a bias of −4 mV. b) The current signal (average current values measured in 1 nm^2^ squares similar to the ones shown in (a)) versus normal force, measured with c‐AFM on a 1L/Bfl/SiC(0001) sample during loading the AFM tip (black squares and curve) and unloading the AFM tip (red circles and curve). The sample was biased at −4 mV. In the inset we show a cartoon of the experimental setup.

To corroborate the finding that under a localized load 1L graphene plus buffer layer undergoes a phase transition to a diamond‐like structure, we used a conductive AFM (c‐AFM) probe to apply a local load and simultaneously measure the electronic current flowing through the AFM tip‐sample contact, see **Figure** **5**. We expect an increase in resistivity (decrease of current) when the load is enough high to activate the graphene‐diamene phase transition. As can be seen in Figure 5, the current increases with increasing normal force (because the contact areas increase with load) until the normal force reaches 175 nN. Then the current decreases with increasing force until reaching the maximal force of 250 nN. The same behavior occurs during the unloading part with a peak at 200 nN. These results demonstrate that for a diamond AFM tip having a radius ≈10 nm, the phase transition occur ≈175 nN. We also remark that this phase transition is metastable. Based on our previous studies and the above reported c‐AFM measurements, diamene formation is pressure‐induced and once pressure is released the created sp^3^ hybridization switch back into sp^2^ hybridization.

## 3 Conclusion

In conclusion, motivated by the recent discovery of a room temperature pressure activated phase transition of epitaxial graphene into a sp^3^ diamene structure, we have investigated the hardness of single crystal SiC coated with epitaxial graphene films. We find that when SiC is coated with 10L of epitaxial graphene, the hardness decreases of ≈88% compared to bare SiC. However, when SiC is coated with a 1 L epitaxial graphene plus a graphene‐like buffer layer, or two graphene layers (in this case the SiC surface is passivated with hydrogen), we observe a dramatic increase in hardness. Precisely, we observe that coating SiC with 1L graphene plus buffer layer can increase the hardness of up to 100% at low loads (between 500 and 900 µN) compared to bare SiC, increasing from 30 to 60 GPa, a value approaching the hardness of diamond. This increase in hardness falls off at high loads, reaching a plateau value of 30% at 10 mN. The experiments also show that the yield point and onset of plastic deformations in SiC increases up to 77% when the SiC surface is coated with epitaxial graphene. These findings shed light on the formation of diamene under the indenter's pressure. In particular, we show that the atomically thin diamene layer can improve hardness of SiC even at high loads, corresponding to indentations of ≈175 nm, i.e., almost 300 times larger than the graphene film thickness. This work opens new possibilities for designing ultra‐hard and ultra‐light thermally conductive coatings for SiC to improve its mechanical performances for applications such as body armors, high‐impact protective coatings, and for use in aeronautics, aerospace, and automobile industry.

## 4 Experimental Section

### Preparation of Epitaxial Graphene Samples

Large area epitaxial graphene films are grown on the Si face of 6H silicon carbide (0001) substrates (II–VI Inc) by the thermal decomposition method.^[^ ^14^ ^]^ The first carbon layer on the Si‐face of SiC is called the buffer layer and this layer is partially covalently bonded to the SiC surface (≈30% sp^3^ bonded carbon atoms^[^ ^27^ ^]^). The layers on top of the buffer layer have carbon atoms in sp^2^ configuration. Therefore, when we refer to single layer graphene, it is a structure comprised of a graphene layer and buffer layer. The quasi‐free standing bilayer graphene is prepared from single layer epitaxial graphene sample by intercalation in hydrogen atmosphere at an approximate pressure of 1000 mbar with hydrogen flow at 3.0 slph. Hydrogen atoms saturate Si—C bonds between SiC surface and buffer layer, which create Si—H bonds and turn sp^3^ bonded carbon atoms into sp^2^ configuration. More details about the growth procedure can be found in Reference.^[^ ^15^, ^16^ ^]^ Schemes of the structures are presented in Figure 1a.

### Nanoindentation Measurements

Nanoindentation experiments are performed on Hysitron TI 950 Triboindenter with a diamond Berkovich tip. The typical radius of curvature of a Berkovich tip, *R*_tip,_ is ≈150 nm at room temperature and in humid atmosphere. The Berkovich tip indents the sample surface at a constant rate until the set maximum load is achieved, then is held for 5 s. The loading time for this process is 62 s. Finally, the tip is unloaded linearly in time within 5 s (see Figure 1c). The used maximal load varied from 300 µN to 10 mN. The sensitivity of measurement is <30 nN and <0.2 nm for load and displacement, respectively.

The unloading part of the load indentation depth curve is analyzed and the hardness *H* and elastic modulus *E* can be determined. The hardness *H* is defined by the following equation

(2) $$H=\frac{P_{\max}}{A_{p}}$$

where *P*_max_ is the maximal applied load and *A*_p_ is the projected area of the indenter in sample surface. For the Berkovich tip, the projected area is equal to

(3) $$A_{p}=24.5h_{c}^{2}$$

Here *h*_c_ is the contact depth which can be calculated from the maximal indentation depth *h*_max_, the maximal applied load *P*_max_, the contact stiffness *S*, and a geometric factor *ε* (for Berkovich tip, *ε* = 0.75) by

(4) $$h_{c}=h_{\max}-\varepsilon \frac{P_{\max}}{S}$$

At large contact depths the ideal tip area function, mentioned in Equation 3 can yield accurate results, whereas at low contact depths the actual tip geometry must be considered to get accurate results, due to the presence of an indentation size effect. The indentation size effect can be caused by several factors, such as the presence of residual stresses and friction/adhesion between the sample surface and the indenter.^[^ ^28^, ^29^ ^]^ We therefore use the following equation to describe the projected area

(5) $$A\;\left(h_{c}\right)=C_{0}\;h_{c}^{2}+C_{1}h_{c}+C_{2}h_{c}^{1/2}+C_{3}h_{c}^{1/4}+C_{4}h_{c}^{1/8}+C_{5}h_{c}^{1/16}$$

The parameters in Equation 5 are obtained through a fitting procedure of the experimental data of the bare SiC (see Figure S5, Supporting Information) with the parameter *C_0_* kept at 24.56, describing the ideal Berkovich diamond indenter.

Contact stiffness *S* is defined as *S = dP/dh*, which can be obtained from the unloading part of a load‐displacement curve as shown in Figures S2–S4 (Supporting Information). Then the reduced elastic modulus *E*_r_ is

(6) $$E_{r}=\frac{\sqrt{\pi }}{2\beta }\;\frac{S}{\sqrt{A_{p}}}$$

where *β* is a correction factor =1.034 for a Berkovich tip. The reduced elastic modulus is given by

(7) $$\frac{1}{E_{r}}=\frac{1-\nu^{2}}{E}+\frac{1-\nu_{i}^{2}}{E_{i}}$$

where *E*_i_ *=* 1140 GPa and *ν*_i_ *=* 0.07 are the known elastic modulus and Poisson's ratio of the indenter, and *E* and *ν* are the same parameters of the sample.

### AFM Nanoindentation Experiments

The AFM nanoindentation experiments are performed on a Bruker Multimode 8 AFM using a diamond AFM probe (Micro Star Technologies, tip radius ≈10 nm, normal spring constant 152 N m^−1^) in ambient conditions. The normal forces between 0 and 200 µN are used to indent a sample's surface. The investigated area is scanned before and after indentation experiment with the same tip in a tapping mode, to distinguish if the residual indentation was created or not.

### Conductive AFM experiments

The conductive AFM measurements are performed with a Bruker Multimode 8 AFM using a sharp conductive diamond probe (Adama innovation, tip radius ≈10 nm, normal spring constant 74 N m^−1^) in ambient conditions. The current dependency on normal load is measured at forces between 0 and 250 nN.

## Conflict of Interest

The authors declare no conflict of interest.

## Supporting information

Supporting Information

## Data Availability

The data that support the findings of this study are available in the supplementary material of this article.
