# Supplementary material for: Giant Increase of Hardness in Silicon Carbide by Metastable Single Layer Diamond‐Like Coating
Source: Adv Sci (Weinh). 2023 Jan 4;10(6):2204562. doi: 10.1002/advs.202204562 (PMC9951309; doi:10.1002/advs.202204562)
Supplement: Supplementary file 1 — Supporting Information [file ADVS-10-2204562-s001.pdf]

Supplementary information for

Giant increase of hardness in silicon carbide by metastable single layer diamond

Nanoindentation Experiments

The Hysitron TI 950 Triboindenter is used to perform nanoindentation experiments in range of loads between 300 µN and 10 mN on a bare silicon carbide sample (SiC(0001)), silicon carbide covered with 1-layer epitaxial graphene plus a buffer layer (1L/Bfl/SiC(0001)), and quasi-free standing bilayer graphene (2L/H-SiC(0001)), schemes of investigated samples are presented in Figure 1a in the main text. First, the indenter is brought close to the sample surface and five indentation spots are selected on the area of 15×15 μm^2. All indentations are further enough from each other, each indentation is performed with different maximal load, and the last indentation, which is located close to the center of selected area, is performed at maximal load of 10 mN. It helps us to locate the area, which was used for indentation experiments, with the

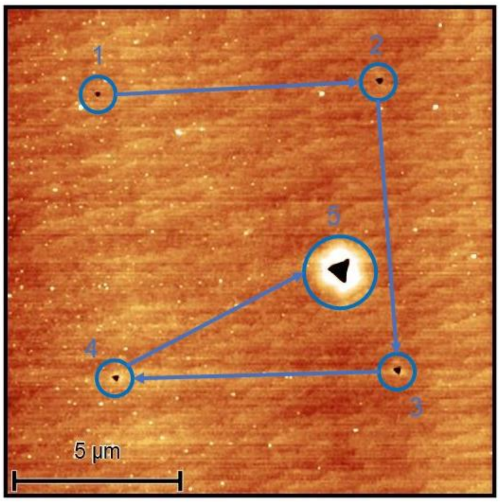

Figure S1: The AFM map of the area after nanoindentation experiments. The circles mark spots where the indentation measurements were performed, and arrows show the experimental proceedings.

AFM tip and move on the surface to locate all indentation spots (see Figure S1).

The force distance curves obtained through nanoindentation experiments are shown in Figures S2, S3 and S4, for bare silicon carbide, 1-layer epitaxial graphene on the SiC silicon face and 2-layers quasi free standing epitaxial graphene on Si-face of SiC, respectively.

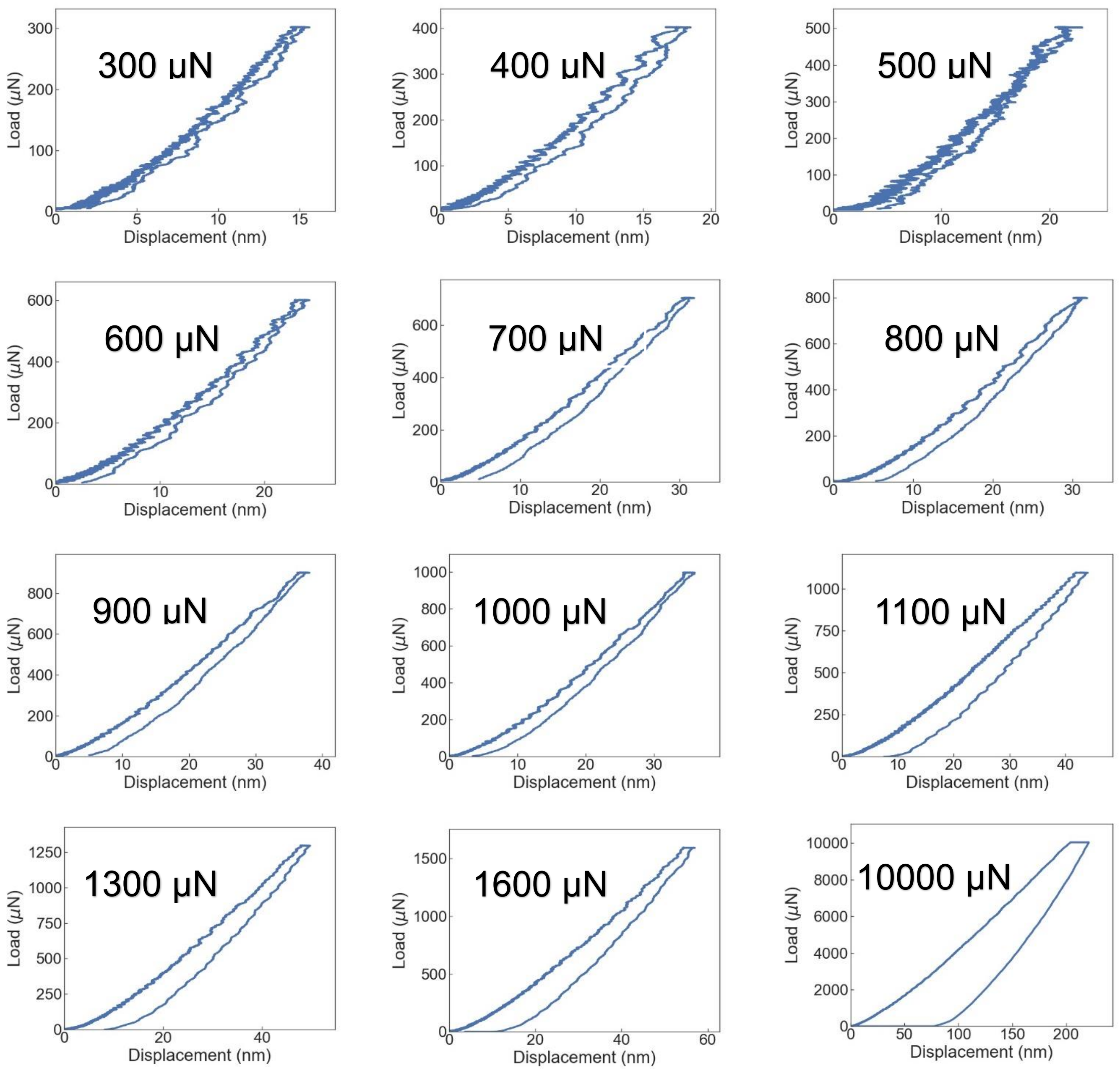

Figure S2: The force distance curves at different maximal load for bare SiC.

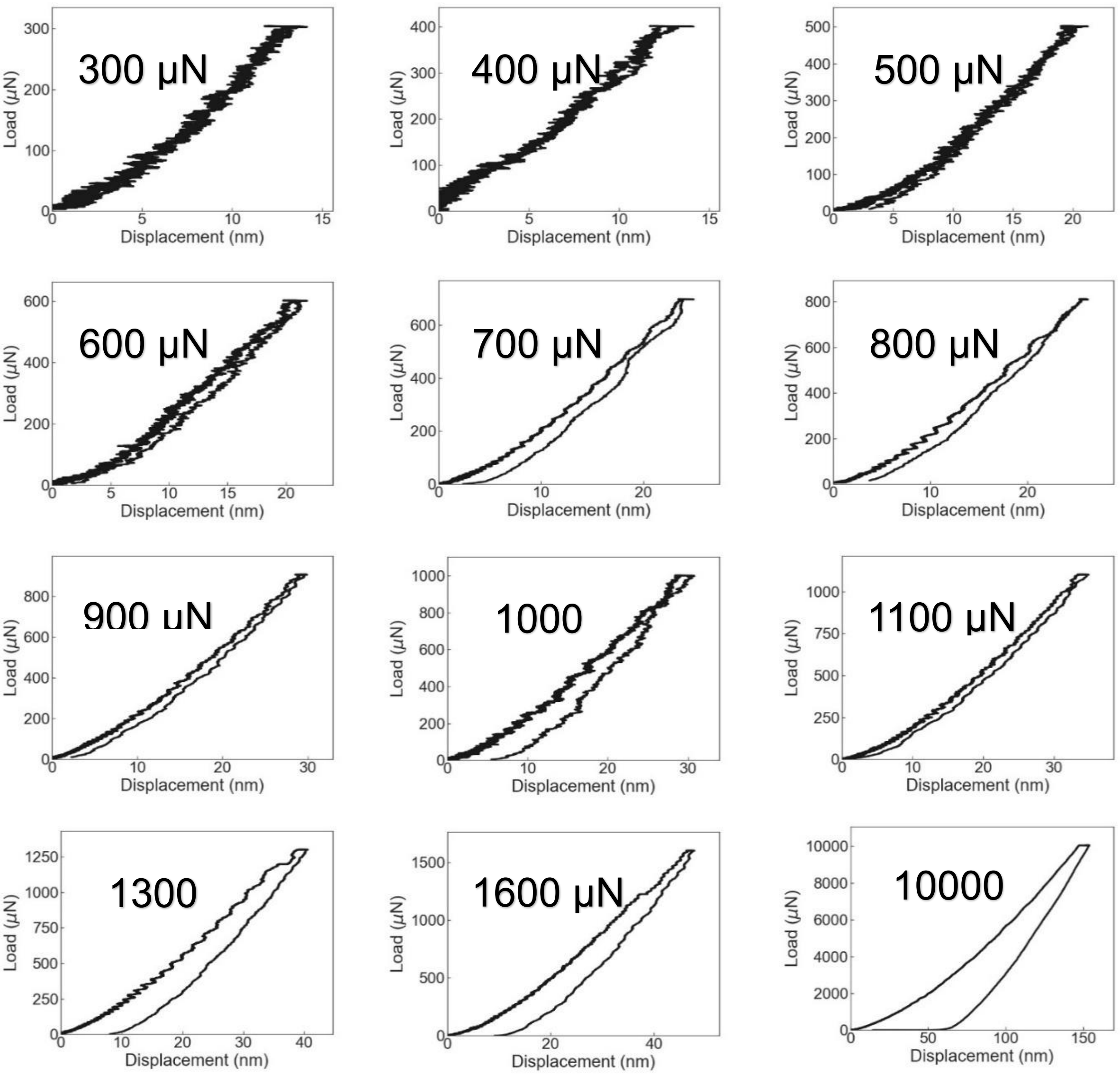

Figure S3: The force distance curves at different maximal loads for 1L/Bfl/SiC.

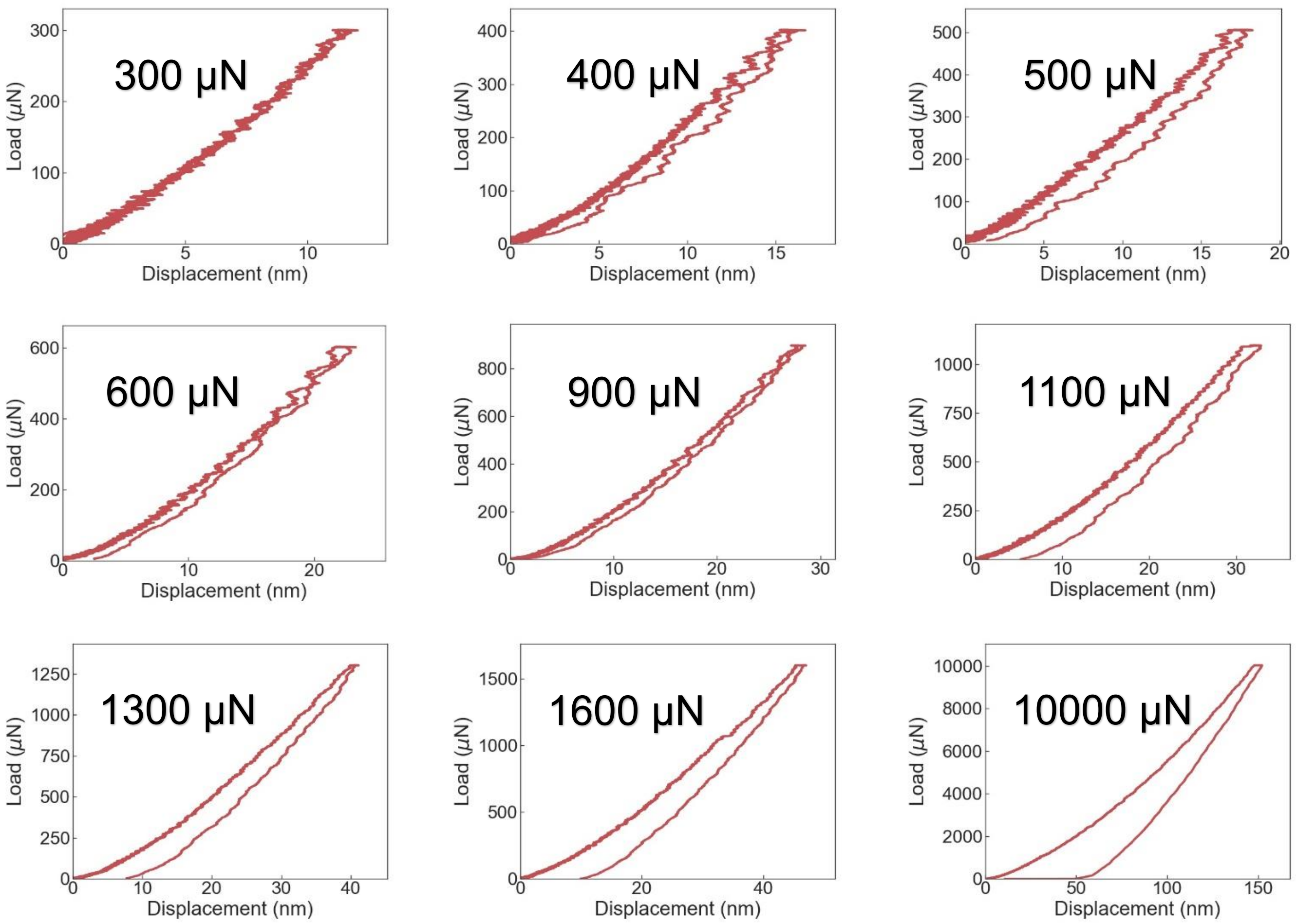

Figure S4: The force distance curves at different maximal loads for 2L/H-SiC.

Indenter shape calibration

We calibrated the Berkovich tip shape using the known hardness of bare SiC (H^{SiC}=30 GPa) and using the equation (5) in the main text. We plotted the contact depth h_c as a function of the maximal load F_{max} divided by the hardness of silicon carbide in Figure S5 and we fitted the data points with the equation (5) to obtain the corresponding coefficients. The coefficient C_0 is kept at 24.56 describing the ideal Berkovich indenter, and the remaining parameters C_1-C_5 are found through the fitting procedure.

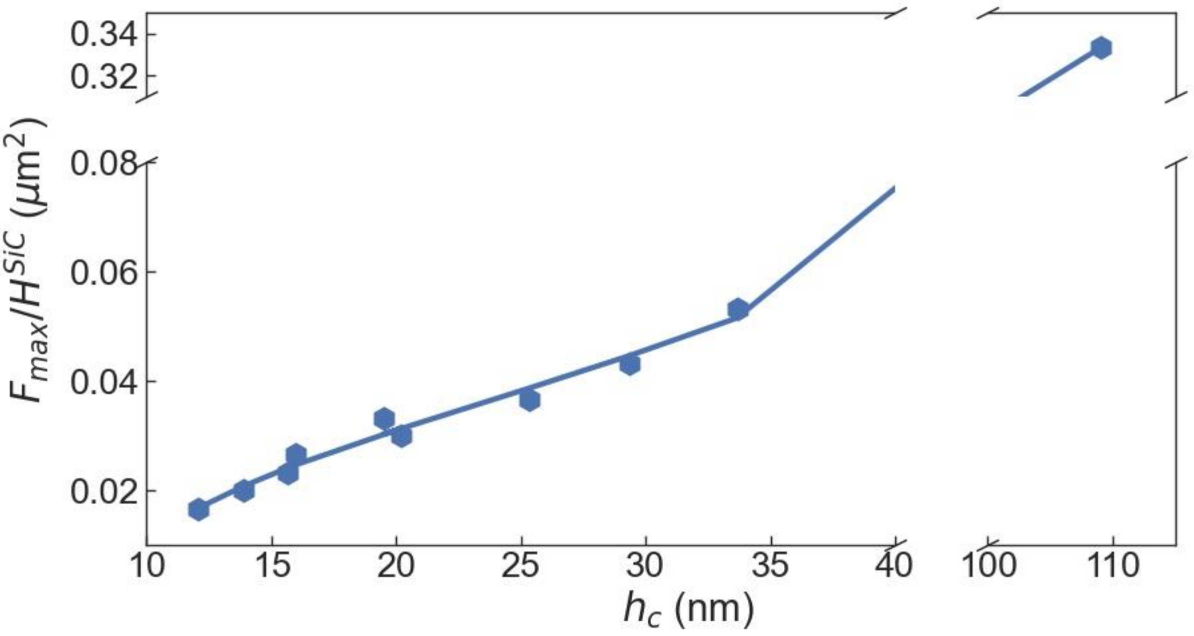

Figure S5: The maximal load divided by silicon carbide hardness (H^{SiC} is 30 GPa) as a function of the contact depth. The markers correspond to experimental data and solid line corresponds to a fit using equation (5) in the main text.

Indentation on 10L epitaxial graphene

We performed indentation experiments on twisted 10-layer (10L) epitaxial graphene grown on the carbon face of SiC(000$\bar{1}$)[1] and we estimated the hardness of 10L graphene using the Oliver-Pharr method. We compared the observed hardness with the hardness of bare SiC (see Figure S6). The results show that the hardness by SiC decreases of 88% when SiC is covered with 10L epitaxial graphene.

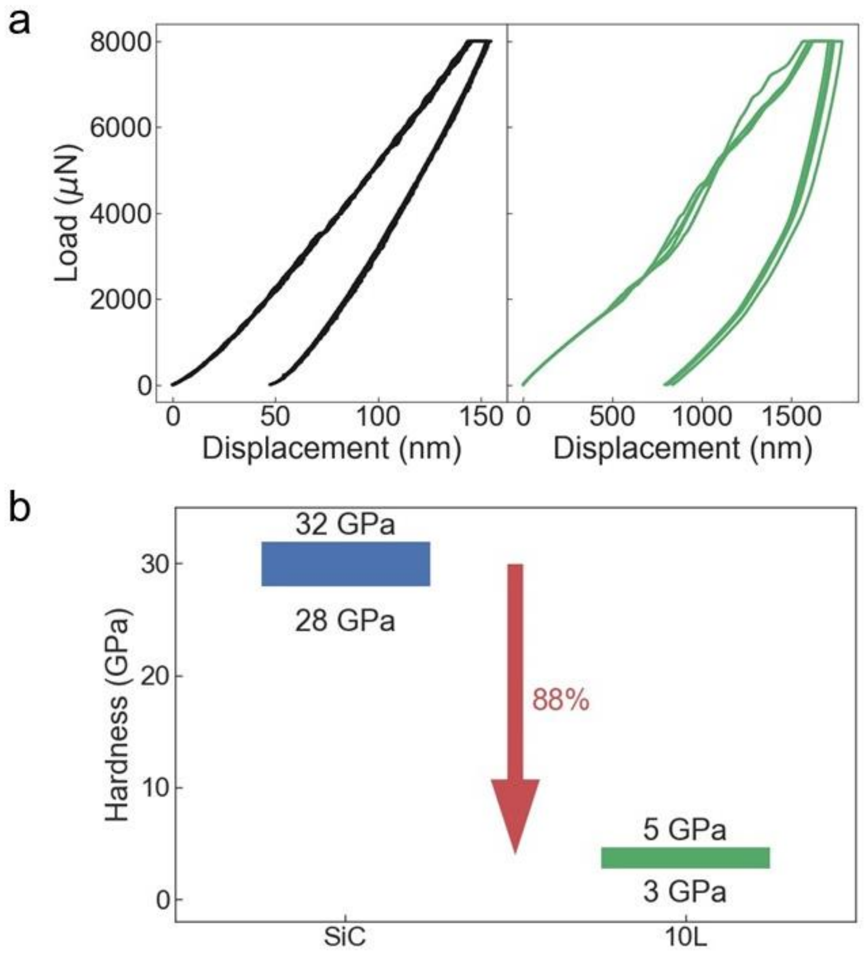

Figure S6: a) The load displacement curves of bare SiC and 10L epitaxial graphene. b) Comparison of hardness between bare SiC and 10L epitaxial graphene on SiC, showing a decrease of 88% in hardness when SiC is covered with 10L epitaxial graphene.

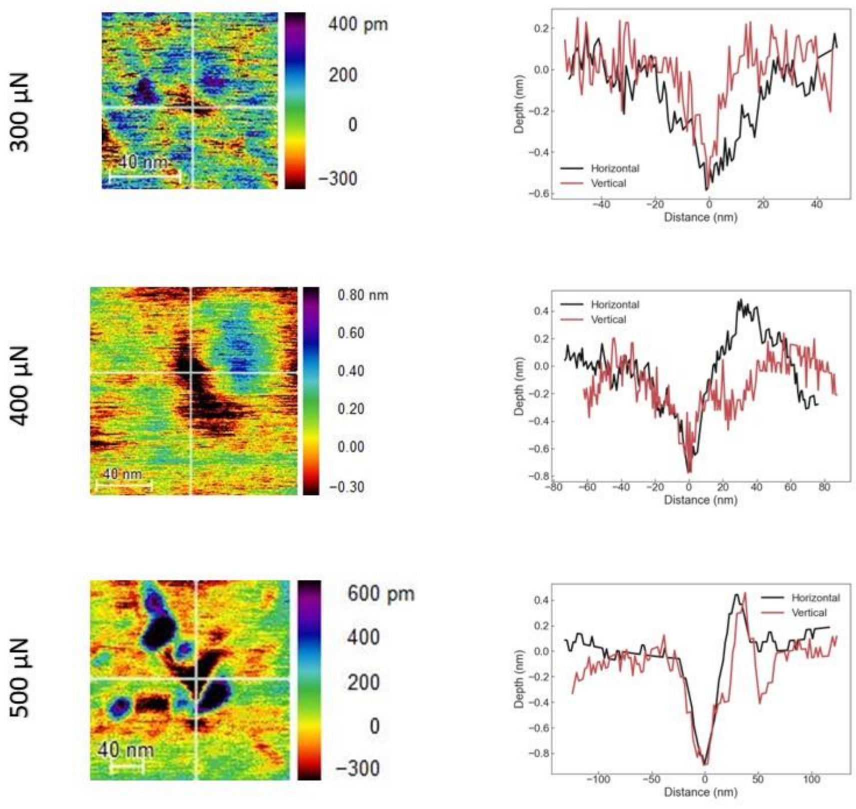

Figure S7: The AFM scans of the residual indentation on bare SiC.

AFM Imaging

We used AFM in tapping mode to obtain the projected area of selected residual indentations and to find the required load to plastically yield the investigated samples. We found residual indentations on the surface of bare SiC for all loads (see Figure S7), which agrees with the load displacement curves (see Figure S2). In case of 1L/Bfl/SiC, we detected the first residual indentation at a load of 500 µN (see Figure S8), which is also in an agreement with load displacement curves (see Figure S3), showing the elastic behavior for loads 300 µN and 400 µN.

These observations were used to calculate the yield point through equation (1) in the main text with tip radius equal to 150 nm.

We estimated the projected area A_p from the AFM scans of residual indentations at loads starting from 900 µN (see Figure S9 and Figure 3a in the main text). This is because from this

load the residual indentation is well defined, and the estimated projected area can be estimated without a large error.

AFM nanoindentation

We performed AFM nanoindentation experiments to create plastic deformation on the investigated samples and compare the effect of the indenter size; in this case the tip radius is 10 nm, with the diamond Berkovich indenter with tip radius of 150 nm. We used a Bruker Multimode 8 AFM setup with a diamond AFM probe from Micro Star Technologies, spring constant is 152 N/m. We first scan the area of interest before the indentation process and after the indentation to observe whether the residual indentation was created. The selected AFM scans after indentation are shown in Figures S10 and Figure S11 with corresponding cross section profiles. The presence of residual indentation as a function of applied load can be found in Figure 4c in the main text. Furthermore, the corresponding estimated yield point is plotted in Figure 4d in the main text.

Finite Element Simulations

We used COMSOL software to perform finite element simulations of an elasto-plastic deformation to investigate stress distribution in bare silicon carbide and silicon carbide covered with 5 Å of diamond. This diamond layer simulates phase transformed graphene into diamene.

The rigid spherical indenter with a radius of 15 nm represents an AFM tip. The sample is prepared as a block with width of 60 nm and height of 40 nm. The mesh prepared before finite element computation is shown in Fig S12a. Elasto-plastical behavior of the sample is described using Voce isotropic hardening model and von Mises stress is used to describe yield function.

The simulations are depicted in Fig. S12 showing the situation at the maximal load which was set at 40 µN (Figs. S12b and 12c), and the final situation of the sample after indentation testing and indenter withdrawal (see Figs. S12d and S12e). The stress at maximal loading is homogenously spread in SiC under the indenter in the case of bare SiC, while in the case of SiC/Diamond the maximal stress is localized in the coating diamond layer and does not propagate too much into SiC. From the simulations after indenter withdrawal, we can see the residual depth of 4.71 nm and 4.57 nm for bare SiC and SiC/Diamond system (see also simulated force distance curve in Fig. S12f), respectively.

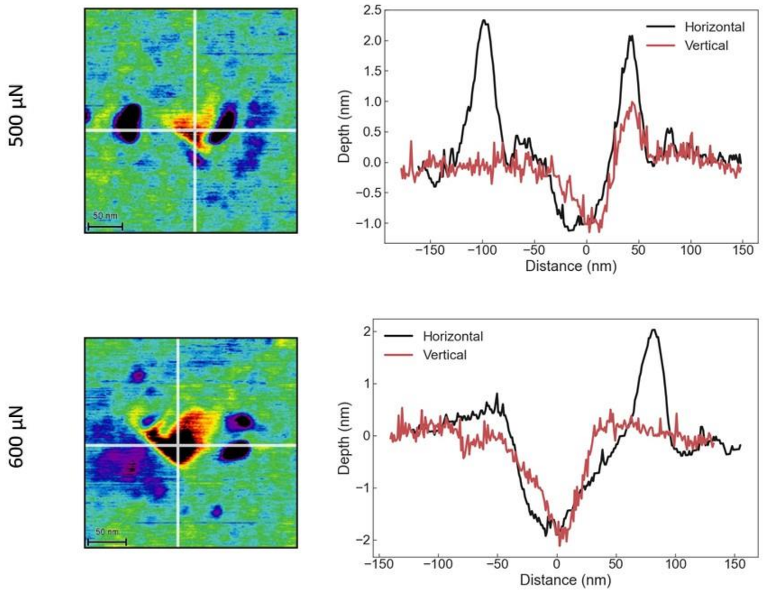

Figure S8: The AFM scans of the residual indentations on 1L/Bfl/SiC(0001).

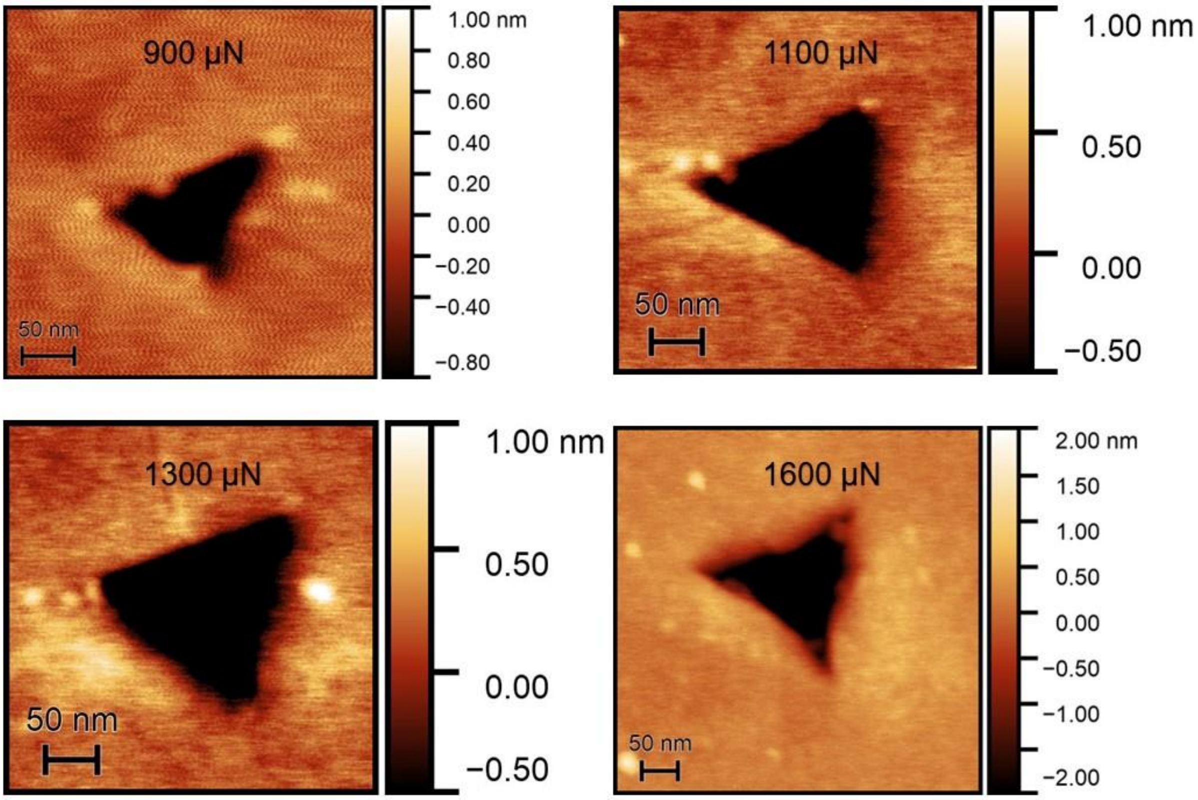

Figure S9: The AFM scans of SiC surface with residual indentations after triboindenter experiments at different loads.

References

1 Hass, J. *et al.* Why multilayer graphene on 4H-SiC(0001[over]) behaves like a single sheet of graphene. *Phys Rev Lett* **100**, 125504, doi:10.1103/PhysRevLett.100.125504 (2008).

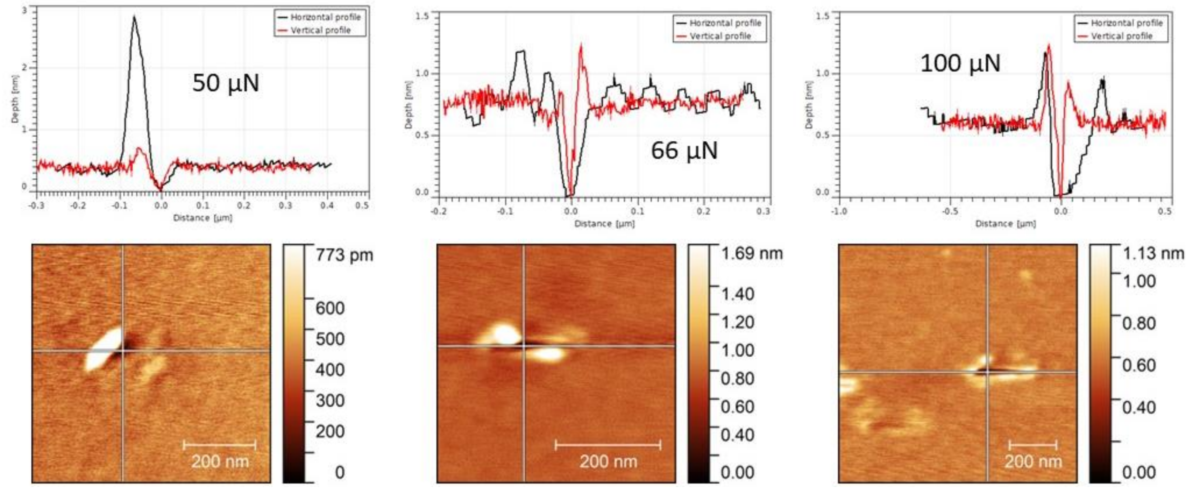

Figure S10: The AFM scans of residual indentations with corresponding cross sections after nanoindentation experiments on a bare SiC sample.

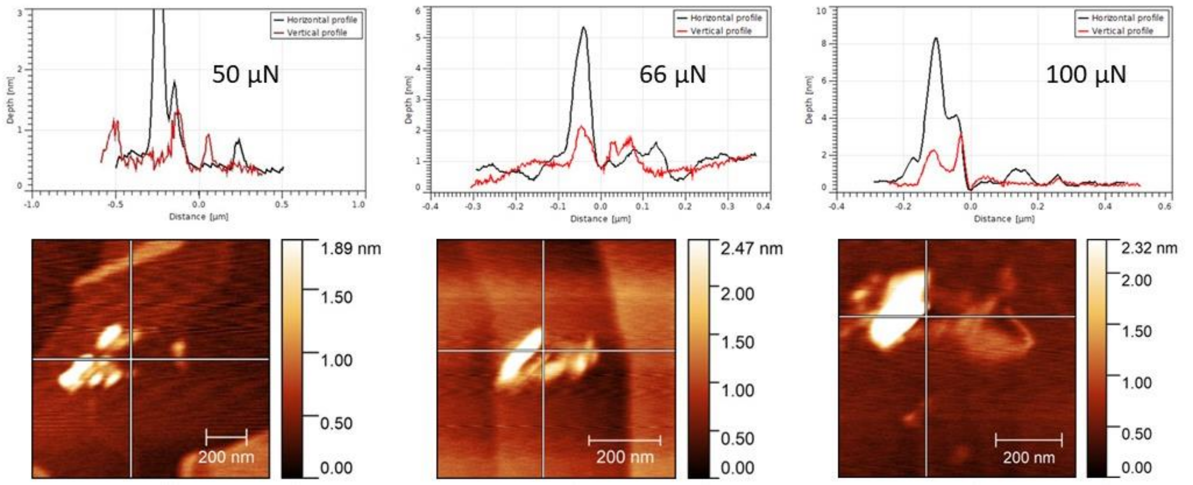

Figure S11: The AFM scans of 1L/Bfl/SiC surface after nanoindentation experiments with corresponding cross sections.

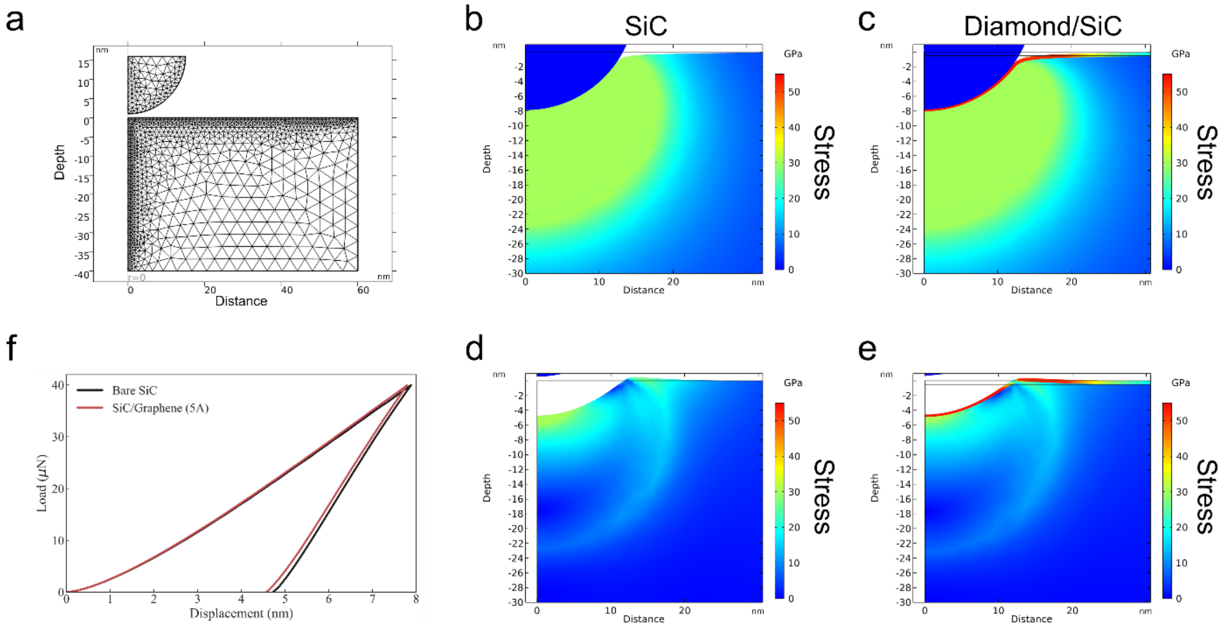

Figure S12: Finite element simulations of the plastic deformation of bare SiC (a, c) and of the composite structure diamond/SiC, where the diamond coating has a thickness of 5 Angstrom (b, d). The maximal applied load is 40 μN as is shown in figures a and b for bare SiC and diamond/SiC, respectively. The final stage of the residual indentations are shown in figures c and d. The color bar represents stress.
